# Application of Skin Electrical Conductance of Acupuncture Meridians for Ureteral Calculus: A Case Report

**DOI:** 10.1155/2011/413532

**Published:** 2011-07-28

**Authors:** Wu-Chou Lin, Yung-Hsiang Chen, Jian-Ming Xu, Der-Cherng Chen, Wen-Chi Chen, Chao-Te Lee

**Affiliations:** ^1^Graduate Institute of Integrated Medicine, School of Chinese Medicine and Graduate Institute of Clinical Medical Science, China Medical University, Taichung 40402, Taiwan; ^2^Departments of Urology, Obstetrics and Gynecology, Neurosurgery, and Medical Research, China Medical University Hospital, Taichung 40402, Taiwan; ^3^Graduate Institute of Geriatric Medicine, Anhui Medical University, Hefei 230000, China

## Abstract

Renal colic is a common condition seen in the emergency department (ED). Our recent study showed that measures of electrical conductance may be used as supplementary diagnostic methods for patients with acute renal colic. Here, we describe the case of a 30-year-old male subject with a left ureteral calculus who presented with frequency and normal-looking urine. He had already visited the outpatient department, but in vain. Normal urinalysis and nonobstructive urogram were reported at that time. Two days later, he was admitted to the ED because of abdominal pain in the left lower quadrant. The urinalysis did not detect red blood cells. Ultrasonography did not indicate hydronephrosis. The meridian electrical conductance and index of sympathovagal balance were found to be abnormal. High level of electrical conductance on the left bladder meridian was found. An unenhanced helical computed tomography was scheduled to reveal a left ureterovesical stone. Ureteroscopic intervention was later uneventfully performed, and the patient's pain was relieved. The follow-up measurements showed that the meridian parameters had returned to normal one month after treatment. This case suggests that bladder meridian electrical conductance might be used as a supplemental method for ureteral calculus diagnosis.

## 1. Introduction

Ureteral calculus-caused renal colic is a common condition seen in the emergency department (ED). Although some patients have unusual or atypical presentations, the most typical clinical feature of acute ureteral colic is a sudden onset of very intense agonizing flank pain that frequently terrifies the patient. Except for pain relief, there are few intervention options for the emergency physician treating an episode of acute renal colic. Most patients are generally recommended a follow-up visit to the outpatient department after pain relief in the ED. The use of ancillary tests, such as ultrasonography and advanced imaging studies, can give the assurance for ureteral obstruction [1]. However, because of the unavailability of the necessary equipment and cost-efficiency, many patients do not receive the above-mentioned examinations in the ED. These patients may suffer from recurrent colicky flank pain during the observation period with possible complications.

Skin electrical conductance, a measure of the skin's conductance between 2 electrodes, is typically measured by small direct current (DC) signal through 2 electrodes on the skin [2]. In traditional Chinese medicine, there are 12 meridians on each the right and left sides of the human body, and skin conductance is found to be significantly higher at acupuncture points [3]. Skin conductance changes with the activity of the subject's autonomic (sympathetic) nervous system [4]. The properties of the meridians can reflect conditions of certain organs by analyzing and comparing their mutual relations with, and changes in, the microelectrical current [5]. The electrical states of the acupuncture points of the human subject are measured by a computerized testing instrument with a very low electrical current. More recently, our report showed that measures of electrical conductance, especially the index of sympathovagal balance, may be used as valuable supplementary diagnostic methods for selective intervention in patients with acute renal colic [6]. 

In this study, we used a device (Meridian Energy Analysis Device, MEAD; MedPex, Taiwan) to measure the meridian electrical conductance and the index of the sympathovagal balance for patients with urolithiasis based on the Ryodoraku theory [5]. This can be easily performed at the bedside within minutes. According to our previous study, the reproducibility of the device is ~93.2%, the average electrical conductance of the renal colic patient group was lower than that of the control group, and the average index of sympathovagal balance of the renal colic patient group was higher than that of the control group. Multiple logistic regression models showed that the index of sympathovagal balance was a significant independent indicator for patients with renal colic to accept urological intervention. The ROC curve in predicting intervention was constructed, and the area under the curve was found to be 0.804 (95% confidence interval [CI], 0.680–0.929) [6]. All procedures were approved by the Ethics Committee on Human Experimentation of the Lin Shin Hospital (Lin Shin IRB no. 000011) in Taiwan. Written informed consent was provided by the patient. In the present study, we tested the concept and hypothesis of whether this intervention option could be used as an auxiliary predictor of the need for intervention for ureteral calculus in the ED.

## 2. Case Report

 A 30-year-old male patient presented to the emergency room with the chief complaint of a dull pain in the left lower lateral abdomen. The onset of pain had been approximately 2-3 days before presentation. He did not have a remarkable past history. He complained of frequency but denied recent trauma or any problems moving his bowels. Although he had visited the outpatient department 2 days before coming to the ED, he was discharged because of normal urine examination and intravenous pyelography results. Because of discomfort with frequency, he visited the ED for medical attention. In the ED, the urine analysis was redone. Similarly, urinalysis did not detect red blood cells in the urine. Serum electrolytes, blood urea nitrogen, creatinine levels, and white blood cell count were normal. Kidney sonography did not show obvious hydronephrosis. Although he did not complain of dysuria, he did mention increased micturation frequency. He did not seem anxious or hypochondriatic, and he went to the toilet several times during observation.

**Figure 1:**
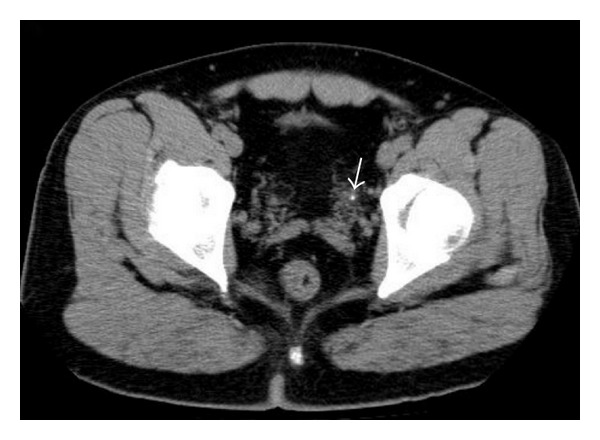
CT showing a small ureteral stone located at the ureterovesical junction area (arrow).

The MEAD device was then used to record the data in the ED. The meridian electrical conductance was low (14.9 *μ*A; normal range, 30–64 *μ*A). The index of sympathovagal balance was high (3.75; normal range, 1.0–1.5). The ratio of electrical conductance of the left side to the right side bladder meridian was obviously abnormal (L/R: 2.09) (Table 1). A urologist was consulted at this time. Unenhanced helical computed tomography (CT) was performed and depicted a tiny calculus at the left ureterovesical junction (Figure 1). The patient was admitted to the urological service and started on an aggressive hydration regimen in an attempt to “flush” the stone. One day later, with his pain worsening, he was taken to the operating room to perform ureteroscopy. A ureteral stone (about 4 mm) was removed, and the patient's pain was relieved (meridian electrical conductance: 14.6 *μ*A; index of sympathovagal balance: 3.31; ratio of electrical conductance of left to right side bladder meridian: 3.26). The subsequent hospital course was uncomplicated. The patient was discharged the following day and instructed to follow up as an outpatient. At the same time, he was encouraged to drink sufficient amounts of fluid to prevent the recurrence of ureteral calculi. The follow-up measurements of the meridian parameters (meridian electrical conductance: 47.8 *μ*A; index of sympathovagal balance: 2.0; ratio of electrical conductance of left to right side bladder meridian: 1.19) returned to normal 1 month after treatment.

## 3. Discussion/Conclusions

 Renal colic is a common condition found worldwide, and it is also one of most common conditions seen in the ED [7]. The majority of patients with ureteral calculus present with moderate to severe flank pain. Although renal colic is often diagnosed by its clinical presentation and physical examination, widespread use of urinalysis, initial plain kidney-ureter-bladder film, ultrasonography, and urogram should be used to confirm and evaluate the presence of ureteral calculus. Hematuria occurs in approximately 90% of patients with ureteral calculus, and 70–90% of urinary calculi are radiopaque [1]. Our patient presented with the symptom of frequency and normal-looking urine, but without renal colic. In our case, the patient did not have a high VAS score, but he had extreme discomfort with urination. The MEAD device can be used as an objective source of further patient information. 

**Figure 2:**
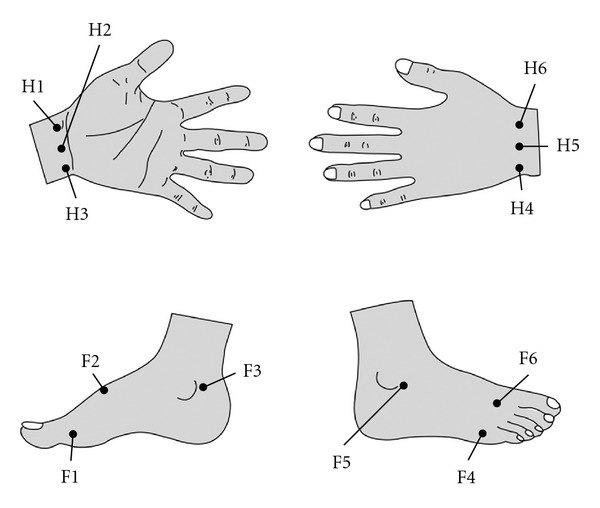
According to the traditional Chinese meridian theory, there are 6 acupoints on each limb (both right and left limbs are symmetric). H1: lung; H2: pericardium; H3: heart; H4: small intestine; H5: triple energizer; H6: large intestine; F1: spleen; F2: liver; F3: kidney; F4: urinary bladder; F5: gall bladder; F6: stomach.

In 1949, a research group headed by the dean of the biology department at Kyoto University found that abnormalities or diseases of the viscera were reflected in measurable changes of bioelectric current. In 1956, after further advancement and study with source points and bioelectric currents, the famous Ryodoraku theory was published [5]. The electrical conductance of 24 acupuncture points (Rydoraku points) in the 12 left meridians and the 12 right meridians were measured. The meridians are as follows: lung (L, H1), pericardium (P, H2), heart (H, H3), small intestine (SI, H4), triple energizer (TE, H5), large intestine (LI, H6), spleen (SP, F1), liver (LIV, F2), kidney (K, F3), urinary bladder (B, F4), gall bladder (G, F5), and stomach (S, F6) (Figure 2). Conductivity at the acupuncture point is directly proportional to the amperage of the DC current that flows through the skin when 12 V are applied to the points one by one. Current is delivered through a thick-wall electrode containing moist cotton wool. Conductance values are calculated and expressed on a scale between 0 and 200. The average conductance value of the 24 meridians in each subject is calculated. For the MEAD analysis, the index of sympathovagal balance is defined as the highest average limb electroconductivity on the dorsal or ventral side, divided by the lowest. All procedures were performed in an air-conditioned environment with the temperature set between 23°C and 26°C. The humidity was also kept constant. Many earlier studies have shown that the nervous system is somehow related to the meridian system [3, 4]. An effectively-functioning body often exhibits effectual conductivity of heat or electrical energy [8]. 

Extensive large-scale consecutive cohorts of patients who visited the ED with ureteral calculus with acute, renal colicky pain are currently enrolled in our clinical trial. Previous studies have shown that renal colic mostly occurs in the early morning (approximately 63%). The subjects in this trial had obvious lower electroconductivity and a higher index of sympathovagal balance. When there is high sympathetic activity in the patient's body, decreased intradermal acetylcholine causes difficulty in the secretion of sweat glands, which in turn leads to a decrease in the electrical conductance in the skin and an increase in resistance. By measuring skin resistance, the function of peripheral autonomic innervation can be indirectly evaluated [9]. The obvious advantage of the neurometer is its noninvasiveness and the fact that testing can be completed in 5 min. Noncontrast helical CT detects obstructing ureteral calculi with a sensitivity of 98%, a specificity of 100%, and positive and negative predictive rates of 100% and 97%, respectively, [10]. In recent years, urinalysis, initial plain kidney-ureter-bladder film, and ultrasonography were still main and effective examinations for renal calculi diagnosis. Because examination with the MEAD device is easy to perform, it might be used to help diagnosing the need for intervention after imaging results in the ED. Nevertheless, the specific relationship between meridian selected, conductance, sympathovagal balance index, and the disease, respectively, need further analyses.

Some studies have shown that up to 40% of patients with urolithiasis have difficulty passing calculi [11]. Urgent intervention is needed in a patient with an obstructed ureteral stone. Delayed intervention will increase the risk of acute pyelonephritis and renal damage. According to this study, the MEAD analysis could provide physicians with additional information by which to judge the patient's condition.

##  Conflict of Interests

All authors stated that they had no conflict of interests.

## Figures and Tables

**Table 1:** The meridian parameters of the patient.

	In ED	After treatment	1 month after treatment	Normal range
Skin meridian electrical conductance (*μ*A)	14.9	14.6	47.8	30–64
Index of sympathovagal balance	3.75	3.31	2.0	1.0–1.5
Left side bladder meridian	35.9	18.9	30.2	N/A
Right side bladder meridian	17.2	5.8	25.3	
Ratio of left to right side bladder meridian	2.09	3.26	1.19	~1
